# N6-methyladenine modification in noncoding RNAs and its function in cancer

**DOI:** 10.1186/s40364-020-00244-x

**Published:** 2020-11-10

**Authors:** Xinyu Yang, Xiang Hu, Jinting Liu, Ruiqing Wang, Chen Zhang, Fengjiao Han, Yuhong Chen, Daoxin Ma

**Affiliations:** 1 Department of Hematology, Qilu Hospital of Shandong University, Jinan, Shandong P.R. China; 2 Shandong Provincial Key Laboratory of Immunohematology, Qilu Hospital of Shandong University, Jinan, Shandong P.R. China

**Keywords:** Noncoding RNAs, m6A modification, Cancer

## Abstract

Non-coding RNAs are the main component of the extensive transcription results of the mammalian genome. They are not transcribed into proteins but play critical roles in regulating multiple biological processes and affecting cancer progression. m6A modification is one of the most abundant internal RNA modification of mammalian cells, and it involves almost all aspects of RNA metabolism. Recent research revealed tight correlations between m6A modification and ncRNAs and indicated the interaction between m6A and ncRNAs act a pivotal part in the development of cancer. The correlation between m6A modification and ncRNAs provides a new perspective for exploring the potential regulatory mechanism of tumor gene expression, and suggest that m6A modification and ncRNAs may be important prognostic markers and therapeutic targets for multiple cancers. In this review, we summarize the potential regulatory mechanisms between m6A methylation and ncRNAs, highlighting how their relationship affects biological functions in cancer.

## Introduction

It is a traditional central dogma that the genetic information presents in the DNA-RNA-protein axis. However, most of the genome sequences are transcribed as noncoding RNAs (ncRNAs), while only 2–3% of the human genome constitutes protein-coding genes [1]. With advances in high-throughput sequencing technologies and computational platforms, thousands of ncRNAs are discovered, such as long noncoding RNAs (lncRNAs), microRNAs (miRNAs), circular RNAs (circRNAs), small nucleolar RNAs (snoRNAs) and so on [2]. According to the length, the ncRNA family is usually divided into two main classes: short ncRNAs and lncRNAs. Short ncRNAs include: miRNAs, siRNAs, snoRNAs, rRNAs, tRNAs, and Piwi-interacting RNAs (piRNAs). In terms of lncRNAs, which are defined as more than 200 nucleotides transcripts, it can be further divided into some subclasses, such as pseudogenes and circRNAs [3]. These ncRNAs act as functional regulatory molecules that mediate cellular processes including chromatin remodeling, transcription, post-transcriptional modifications and signal transduction. The networks in which ncRNAs engage can influence specific cellular biological responses in multiple developmental and pathological contexts, such as the pathogenesis of cancer [4]. Increasing evidences reveal that ncRNAs may be promising biomarkers and potential targets for diagnosis and treatment of cancer, which are tightly associated with the proliferation, metastasis and chemoresistance of cancer cells [5].

Eukaryotic ncRNAs undergo a range of post-transcriptional modifications, increasing the diversity of the transcriptome without requiring increases in genome size. The most widespread post-transcriptional modification of ncRNAs contains A-to-I RNA editing, pseudouridylation and N6-methyladenosine (m6A) [6–8]. m6A modification, first discovered during the 1970s, is a dynamic reversible modification that can be catalyzed or removed by specific mammalian enzymes. As one of the most abundant internal RNA modification of mammalian cells, m6A modification is involved in almost all aspects of RNA metabolism [9].

Recent research shows that m6A modification is not only ubiquitous in mRNA, but also widespread in ncRNAs [10]. Emerging evidences indicated that the interaction between m6A and ncRNAs act a pivotal part in the development of cancer [11]. In this review, we summarize the potential regulatory mechanisms between m6A methylation and ncRNAs, highlighting how their relationship affects biological functions in cancer.

## m6A modification

**Fig. 1:**
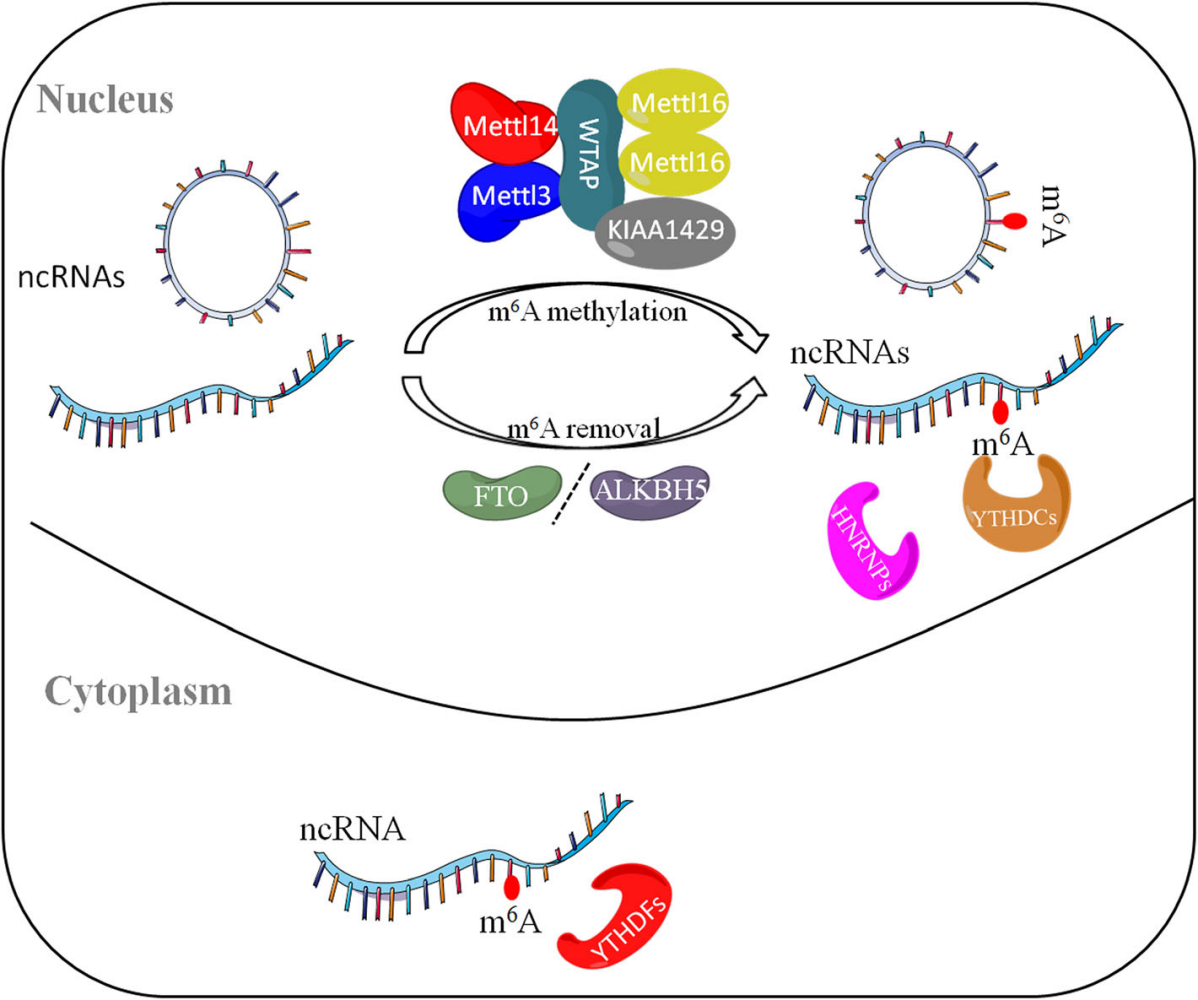
The reversible process of m6A modification in ncRNAs. The process of m6A methylation on ncRNAs involves three crucial components: m6A methylase, m6A demethylase and m6A methylation recognition protein. m6A writer complex composed of the METTL3-METTL14-WTAP core component and other regulatory cofactors. The m6A modification can be removed by m6A demethylase enzymes ALKBH5 and FTO. M6A modifications are functionally facilitated by the m6A methylation recognition protein YTHDF1, YTHDF2, YTHDF3, YTHDC1, YTHDC2 and HNRNP proteins

m6A is enriched in many eukaryotic species of mammals, plants, and yeast [9]. m6A modification mainly occurs in common RRACH (R = G or A, H = A, C or U) sequences. It was found by high-throughput sequencing that m6A was not randomly distributed, but was clustered in the precursor mRNA and the termination codon, 3′ non-translation region (3'UTR), and inner long exon of matured mRNA [12, 13]. It has now become clear that m6A also exists on most ncRNAs, including ribosomal RNAs (rRNAs), lncRNAs, miRNAs, small nuclear RNAs (snRNAs), and circRNAs. The process of m6A methylation involves three crucial components: “writers”, “erasers” and “readers” [14]. They can respectively add, remove, or preferentially recognize the m6A site and thus alter important biological functions [15]. The m6A catalyzing complex “writers” contains a heterodimeric complex of methyltransferase-like protein (METTL) 3, METTL14, KIAA1429, WTAP and a homodimeric complex of METTL16. METTL16 mediates the m6A modification of multiple ncRNAs including the U6 snRNA, numerous lncRNAs and pre-mRNAs [16]. The m6A “erasers” include two demethylase enzymes: AlkB homolog 5 (ALKBH5) and fat-mass and obesity associated protein (FTO), while FTO also exhibits demethylase activity toward other types of modification on RNA [17, 18]. The m6A “readers” are consisted of the human YTH domain family (YTHDF) 1, YTHDF2, YTHDF3, the human YTH domain containing (YTHDC) 1, YTHDC2 and the heterogeneous nuclear ribonucleoprotein (HNRNP) proteins [14, 19]. The writers and erasers cooperatively determine the distribution of m6A modification on RNA, whereas the readers mediate m6A-dependent regulation on RNA metabolism and function. They play a vital regulatory role in the expression of the entire genome, and have great effects on the normal physiological functions or pathological diseases [20] (Fig. 1).

m6A is important for apparent transcriptional regulation, and involved in nearly every stage of RNA metabolism, including RNA processing, nuclear output, translation and RNA degradation [21, 22]. METTL16 catalyzed m6A modification at position 43 of U6 snRNA, which is important for the 5′-splice site recognition by U6 snRNA during pre-mRNA splicing. m6A reader protein hnRNPG can recognize the m6A adjacent splice sites and recruit the RNA polymerase II to promote exon inclusion in the RNA splicing [16]. For the nuclear output, METTL3, WTAP are reported to be associated with the TREX complex, which mediates the export pathway. In addition, knockdown of YTHDC1 results in the mRNA export defect of certain transcripts. For the translation and degradation, YTHDF1 modulates the subcellular distribution and translation status of the m6A-modified mRNA by recruiting the eukaryotic initiation factors 3(eIF3), while YTHDF2 mediates the transfer of the bound RNA from the translatable pool to RNA decay sites. On the top of that, recent studies have found m6A modifications can also regulate phase separation potential of mRNA [8].

Recent studies revealed that most of the writer, eraser and reader proteins are dysregulated in multiple cancers, suggesting the involvement of m6A system in the tumorigenesis. For instance, the METTL3 and METTL14, highly expressed in acute myeloid leukemia (AML), play a critical role in leukemia progression through promoting translation of target mRNAs, including MYB, MYC, BCL2, and PTEN [23]. Unexpectedly, the FTO is also highly expressed in AML cells and acts as oncogene to promote leukemogenesis and inhibit all-trans-retinoic acid (ATRA)-mediated leukemia cell differentiation by destabilizing ASB2 and RARA mRNA via reducing m6A abundance on these mRNA transcripts [24]. These studies suggest the impact of m6A modification on tumor may be dependent on the expression and function of certain target RNAs. As recent studies reveal ncRNAs can regulate cell proliferation and metastasis, stem cell differentiation and homeostasis in cancer, it is worth more investigation on the role of the m6A modification of ncRNAs in the pathogenesis and development of cancers.

## Non-coding RNAs

NcRNAs are the main component of the extensive transcription results of the mammalian genome [2]. NcRNAs are not transcribed into proteins but play roles as master regulators of gene expression in normal physical function and disease [25]. Non-coding RNAs can also be divided into housekeeping ncRNAs and regulatory ncRNAs (regulatory ncRNAs) according to expression and functional characteristics. The former one includes: rRNAs, tRNAs, snoRNAs, small nuclear RNAs (snRNAs) and telomerase RNAs, and is necessary for cell survival. The latter one involves lncRNAs, microRNA, small interfering RNAs (siRNAs), piwi-interacting RNAs (piRNAs), promoter-associated transcripts (PATs), enhancer RNAs (eRNAs), and circRNAs, and it regulates transcription and translation processes [3]. NcRNAs serve as potential biomarkers for the diagnosis, prognosis, and clinical treatment of cancer patients [26, 27].

### LncRNAs

LncRNAs are defined as transcripts greater than 200 nucleotides with no or limited coding protein capacity [28]. Most lncRNAs have several similar features compared to coding mRNAs: they are both 5′ capped, spliced and polyadenylated. Genomic locations define lncRNAs into three subtypes: intergenic lncRNAs, intronic lncRNAs and antisense lncRNAs. LncRNAs are also involved in many molecular and cellular processes, such as transcription, splicing, translation, chromosomal dosage compensation and so on [29, 30]. With the emergence of their various functions, lncRNAs have been proven to play many crucial roles in various kinds of diseases by regulating gene expression at different transcription levels [31].

Currently, researchers have described the important functions and complex mechanisms of lncRNAs. From the perspective of gene regulation, lncRNAs have the following advantages: a. They can easily combine with homologous DNA sequences (genes that transcribe lncRNAs and genes with similar sequences). b. They can easily bind to homologous RNA sequences. c. Their ribbon-like characteristics can be folded into complex secondary structures, which can easily bind to a variety of proteins [32].

LncRNAs can participate in the regulation of various biological processes by interacting with protein, DNA and RNA [33]. LncRNA pncRNA transcribed from the cyclin D1 promoter was found to specifically bind to the RNA binding protein TLS (Translocated in liposarcoma), thereby inhibiting histone acetyltransferase (HAT) activity to exert transcriptional repression [34]. LncRNA pRNA interacts with the target site of the transcription factor TTF-I to form a DNA:RNA trimer, which mediates the recruitment of DNMT3b and the silencing of rRNA genes [35]. It has been found that MALAT1, another lncRNA, have the sponge adsorption function, which promotes tumor cell proliferation and metastasis through sponging many miRNAs [36].

### miRNAs

miRNAs are short non-coding RNAs around 22 nucleotides, and they can endogenously express and regulate mRNA post-transcriptional gene expression by binding to the miRNA response elements (MREs) on their target transcripts. They have a variety of important regulatory effects in cells [37]. Currently, miRNA mediated mRNA degradation is considered to be one of the most basic regulatory epigenetic mechanisms which regulates several key biologic processes (e.g. signal transduction, cell proliferation and differentiation, and apoptosis). miRNAs reduce gene expression by binding to regions of 3′ untranslated mRNAs, through mRNA degradation or inhibition of translation. The relationship between gene expression and miRNA is usually complicated: a single miRNA can target multiple genes and a single gene may be targeted by multiple miRNAs [38].

Since miRNAs could regulate multiple genes, it is not surprising that many miRNAs have been found play important roles in various symptoms, such as tumor, immunological and inflammatory diseases [39]. In pancreatic ductal adenocarcinoma, dys-regulated miRNAs promote the proliferation, invasion and apoptosis of cancer cells by affecting the expression of genes involved in signal transduction of cancer cells [40]. miRNAs play key roles in maintaining immune homeostasis. Studies have found that Dicer-deficient T cells or B cells cause miRNA expression levels to be reduced, and the imbalance of miRNAs is strongly related to the development of autoimmune diseases [41, 42]. Many miRNAs are also differentially expressed in autoimmune diseases such as Rheumatoid arthritis (RA), systemic lupus erythematosus (SLE) and inflammatory bowel disease (IBD), which indicates that miRNAs can be used potentially as a biomarker or treatment of autoimmune diseases [43–45]. With the discovery of cellular vesicle transport mechanisms, exosomes have pushed miRNA research to a new climax. As an important medium that mediates communication between cells, many miRNAs have been found in exosomes [46]. The study found that exosome-mediated miRNA transfer may also serve as the messenger role in intercellular communication and participate in the exchange of genetic material between cells.

### Circular RNAs

Circular RNAs, unlike traditional linear RNAs, are unusual forms of RNA molecules, which have a closed loop structure without a “hat” structure at the 5' end and a polyadenylation tail structure at the 3' end. Therefore, the circRNAs are not affected by exonucleases, and their expression are more stable and difficult to degrade. The main function of circRNAs is the adsorption of miRNA sponge, which is known as the competing endogenous RNAs (ceRNA) mechanism. When the miRNAs are absorbed, they are deactivated from their target genes and target genes can be regulated at the transcription level [47].

There are several biological functions of circRNAs: multiple circRNAs function as “sponges” for miRNAs; some circRNAs can “sponge” other factors, such as RNA binding proteins; largely expressed circRNAs may have other unknown regulatory functions; circRNAs have the function of protein translation [48, 49]. CircRNAs mainly function is sponge adsorption of miRNAs: circRNAs molecules are rich in miRNA binding sites. By adsorbing miRNAs, circRNAs can suppress the inhibitory effect of their target genes and regulate target genes at the transcription level. The circRNA-miRNA-mRNA axis plays an important role in cellular physiology functioning, cancer progression, and immune responses [50, 51], suggesting that circRNAs have potential value in disease diagnosis and treatment.

The expression levels of circRNAs are related to the clinicopathological characteristics of cancer patients. Increased evidence suggests that circRNAs may become a useful molecular biomarker for human diseases since they have strong stability and are hard to be degraded due to their closed structure [52].

## m6A modification in different non-coding RNAs

### m6A modification and lncRNAs

#### The role of m6A modification in lncRNAs

The m6A methylation landscape of human lncRNAs is essentially different from that of mRNA. It is reported that the average enrichment scores of lncRNAs were similar to those of mRNA in cell lines, but the number of m6A peaks distributed in lncRNAs is significantly lower than those in mRNAs [53]. In human fetal tissue, lncRNAs were less methylated than mRNAs with an average of 21%. The distribution of m6A on lncRNAs transcripts is roughly uniform with a slight increase at the 5′ end, and the most frequent consensus motifs were GG/A(m6A) CH [54].

The local structure of lncRNAs can be altered to promote their combining with RNA binding protein (RBP) by m6A modification and further influence some important biological processes. It is confirmed that m6A modification on the metastasis-associated lung adenocarcinoma transcript 1(MALAT1), a highly expressed nuclear lncRNA, is able to affect the localization and activity in the nucleus of MALAT1 and regulate the protein binding through a change in RNA structure [55]. m6A methylation occurred in the region of the lncRNA MALAT1 clip structure, which resulted in the weakening of the U-A pairing effect and loosened the structure of the hairpin. It can in turn enhance the combination of the heterogeneous nuclear ribonucleoprotein C (HNRNPC) and the lncRNAs [56]. As m6A “readers” could recognize the m6A modification, the function of m6A modified lncRNAs can be affected by interacting with m6A “readers”. Studies have shown that m6A methylation plays a significant role in lncRNA XIST-mediated silencing or X chromosome genes inactivation. The methylation-recognition protein YTHDC1 binds to the m6A methylation region on XIST, and recruits silent proteins to complete the entire gene suppression process [57].

m6A is a positive mediator in the ceRNA system, which can modulate the interaction of lncRNA-miRNA. Recent studies have shown that the m6A modification of linc1281 can regulate the differentiation of mouse mESCs by sequestering pluripotency-related let-7 family miRNAs. The m6A-deficient A-G mutant inhibits the interaction between linc1281 and let-7 miRNAs, and only linc1281 with m6A modification can directly bind let-7 miRNAs [58]. However, the distribution characteristics and functions of m6A on lncRNAs are not very clear at present, thus applying further research is essential.

#### The role of m6A modification in lncRNAs in tumor

Although only a small part of the lncRNAs in the genome have been characterized for their functionalities, more and more studies found that m6A and lncRNAs are related to the biological behaviors of cancer, such as enhancing the stemness-like properties of cancer cells, promoting the growth and proliferation of cancer cells, and increasing anti-radiotherapy or chemotherapy [59].

Recently, numerous studies have demonstrated that m6A modification can directly regulate the stability of lncRNAs to affect the development and metastasis of tumors. For instance, the m6A demethylase ALKBH5 inhibits the progress of pancreatic cancer by demethylating lncRNA KCNK15-AS1 and increasing the stability of KCNK15-AS1, and down-regulation of KCNK15-AS1 inhibited the cell migration and invasion [60]. In addition, IGF2BP2 can regulate the stability of lncRNA DANCR by serving as an m6A reader to promote the cell proliferation, stemness-like properties and tumorigenesis of pancreatic cancer cells [59]. Plasmacytoma Variant Translocation 1 (PVT1) is a well-known oncogenic lncRNA. ALKBH5 can bind to PVT1 to inhibit its degradation and promote its stability. The high expression of PVT1 promotes the OS cell proliferation, migration and invasion [61]. In head and neck squamous cell carcinoma (HNSCC) cells, the stability of LNCAROD is enhanced through the METTL3 and METTL14-mediated m6A modification. The high expression of LNCAROD promotes cancer progression by facilitating the formation of a ternary complex with HSPA1A and YBX1 [62]. It is found that Hippo/YAP signaling is closely related to the colorectal cancer progression. YTHDF3 is a key target in YAP signaling pathway. It can promote m6A-modified lncRNA GAS5 degradation, and m6A-modified lncRNA GAS5 can regulate YAP phosphorylation activation. These three elements form a negatively regulated functional loop lncRNA GAS5-YAP-YTHDF3 axis in the progress of CRC [63].

LncRNAs can also play a vital role in the development and growth of tumors by the sponge adsorption function. The lncRNA FAM225A has a highly enriched m6A modification site, and the METTL3-mediated m6A modification can enhance the stability of FAM225A. The high expression of FAM225A can act as a ceRNA that binds miR-590-3p and miR-1275 to upregulate the expression of ITGB3, which promotes the proliferation, migration, invasion, tumor growth and metastasis of nasopharyngeal carcinoma cells [64]. In hepatocellular carcinoma (HCC), METTL3-mediated m6A modification enhances the stability of LINC00958 transcript, which can upregulate hepatoma-derived growth factor (HDGF) expression by sponging miR-3619-5p to promote tumor growth [65]. In non-small-cell lung cancer (NSCLC), the METTL3-YTHDF3-MALAT1-miR-1914-3p-YAP signaling axis plays a crucial role in the invasion and metastasis of tumor. METTL3/YTHDF3 complex can increase the stability of MALAT1. In addition, as a competitive endogenous RNA, MALAT1 acts on miR-1914-3p, and miR-1914-3p can decrease YAP expression by binding the 3'UTR of YAP, which increases YAP expression and activity and further promotes NSCLC drug resistance and metastasis [66].

LncRNAs also play important roles in carcinogenesis via regulating the interaction between m6A-related protein and RNA. As a key cell-cycle molecule, the oncogene FOXM1 plays crucial roles in glioblastoma stem-like cells (GSC) proliferation, self-renewal and tumorigenesis. LncRNA FOXM1-AS facilitates the interaction between ALKBH5 and FOXM1 nascent transcripts and induces the demethylation of FOXM1 pre-mRNA, which increases the expression of FOXM1 and promotes the progression of glioblastoma [67–69]. LncRNA GATA3-AS is transcribed from the antisense strand of the GATA3 gene. It can be used as a cis-acting element that KIAA1429 targets m6A to regulate GATA3 pre-mRNA. Under the induction of lncRNA GATA3-AS, the m6A methylation on GATA3 pre-mRNA 3'UTR is preferentially induced by KIAA1429 in liver cancer cells, which promotes the degradation of GATA3 pre-mRNA. The down-regulated expression of GATA3 promoted the tumor cell proliferation and metastasis [70]. The lncRNA growth arrest special 5 (GAS5) exerts suppressive effects on many kinds of cancers [71]. For example, in cervical cancer (CC), lncRNA GAS5-AS1, the antisense RNA of GAS5, interacted with RNA demethylase ALKBH5 to decrease m6A modification of GAS5 and increase the stability of GAS5 by the YTHDF2-dependent degradation pathway. The high expression of GAS5 significantly reduced the proliferation, migration, and invasiveness of CC cells [72].

In conclusion, these findings have confirmed the clinical significance of lncRNAs and m6A in tumorigenesis and development. They provide new indicators and promising therapeutic targets for tumor prognosis, but are far from specific clinical applications.

### m6A modification and miRNAs

#### The role of m6A modification in miRNAs

Analysis of the MeRIP-Seq data set revealed a strong correlation between m6A and miRNA binding sites. It was reported that m6A modification in mRNAs are enriched in 5′ UTRs, around stop codon and in the proximal region of 3′ UTRs [12]. Moreover, researchers found that miRNAs-targeted sites were also located at the 5′ end and 3′ end of 3′ UTRs, which suggests the potential relationship between m6A and miRNA [73]. The presence of binding sites suggests a closely interaction between mRNA methylation and miRNAs. It will be important to determine whether adenosine methylation contributes to the effect of miRNA-induced mRNA silencing.

m6A modification was identified on the primary miRNAs (pri-miRNAs), and affected the biosynthesis of miRNA. The first step in miRNA biosynthesis is the processing of pri-miRNA with the help of a microprocessor. The microprocessor consists of the RNA-binding protein DGCR8 and type III ribonuclease Drosha [74]. Precursor miRNA (pre-miRNA) was firstly produced with the help of DGCR8 to recognize the junction of the stem and flanking single-stranded RNA on the pri-miRNA hairpin, then Drosha was recruited to cleave the double-stranded RNA [75, 76]. A series of rigorous experiments proved that m6A methylation modification is a new type of regulation method involved in miRNA biosynthesis. METTL3 has been shown to be capable of adding m6A modification of adenylate (A) of GGAC motif on pri-miRNA. Thus, the RNA methylation modification promotes the specific recognition of pri-miRNA by DGCR8, thus becoming a very important marker in the process of miRNA mature body processing [10]. Therefore, researchers believe that m6A is a very important RNA marker which mediates the activation of downstream microprocessors, initiating a series of processes such as pri-miRNA to pre-miRNA and mature miRNA.

m6A modification affects the maturation of miRNA. As the “reader” of m6A modification, the RNA-binding protein HNRNPA2B1 could bind m6A-modified RNA in vitro and in vivo. It was confirmed that HNRNPA2B1 recognized the m6A on pri-miRNA, and activated the pre-miRNA processing pathway, then affected the processing of mature body. HNRNPA2B1 bound to the m6A modification in the pri-miRNA transcript subset, interacts with the miRNA microprocessor complex protein DGCR8, and promotes pri-miRNA processing. Deletion of HNRNPA2B1 and depletion of METTL3 also cause similar processing defects in these pri-miRNA precursors [77].

Study has found that miRNAs can in turn regulate m6A methylation. Recent evidence suggests that miR-145 can regulate m6A levels by targeting the 3'UTR of YTHDF2 mRNA in HCC cells. YTHDF2, as a “reader” protein of m6A, recognizes and degrades mRNA with m6A modification, and the miR-145 levels were inversely related to YTHDF2 mRNA levels [78]. The relationship between m6A and miRNA is very close, but the specific regulatory mechanisms need to be discovered in the future.

#### The role of m6A modification in miRNAs in tumor

Recent studies have found that m6A modification of miRNAs is closely related to tumorigenesis, development and metastasis [79]. miRNAs have been shown to regulate many cellular processes in animals through post-transcriptional regulation of gene expression. The abnormal expression of miRNAs mediated by m6A modification helps certain activation of oncogenes or tumor suppressor genes, and plays a key role in the occurrence and development of tumors.

The m6A modification could affect the development of cancer by promoting the process and maturation of miRNAs. In rectal cancer, researchers have revealed that up-regulation of METTL3 can promote the maturation of pri-miR-1246 by methylating pri-miR-1246. miR-1246 further inhibits the expression of the tumor suppressor gene SPRED2 to promote cancer cell migration and invasion [80]. In HCC, especially in metastatic HCC, it has been shown that the expression of METTL14 in tumor cells and tissues were reduced, which is closely related to the poor prognosis of liver cancer. Further research confirms that METTL14 can positively regulate the processing of pri-miR126 by modulating its interacting with DGCR8. As a tumor suppressor gene, miR126 is a target gene of METTL14 in liver cancer metastasis. Therefore, METTL14 has an important role in inhibiting metastasis of hepatocellular carcinoma by regulating pri-miRNA [81]. The interaction between METTL14 and miRNA signaling might offer a possible therapeutic target of HCC. It has also been reported that m6A-modified pri-miR126 processing can even activate the PI3K-AKT-mTOR pathway, which plays an important role in the development of pulmonary fibrosis in rats [82]. In pancreatic cancer, it has reported that the deposition of cigarette smoke condensate caused the hypomethylation of METTL3 promoter and induced METTL3 highly expressed. It increases the m6A modification of the pri-miR-25, and induces the overexpression of mature miR-25. Mature miR-25 inhibits the expression of its target gene PHLPP2, thereby activating the AKT signaling pathway and promoting the occurrence and development of cancer [83].

miRNAs in turn can regulate m6A methylation to promote the progression of cancer. In HCC, miR-145 expression level is negatively correlated with YTHDF2, and overexpressed miR-145 lead to decreased YTHDF2 expression. Combined the predicting result that miR-145 targets the 3'UTR of YTHDF2, miR-145 regulates m6A levels to inhibit the proliferation of HCC cells [78]. METTL3 was reported to promote human lung cancer cells proliferation, survival, and invasion by enhancing the translation of important oncogenes such as EGFR and TAZ. The researchers confirmed that the expression level of miR-33a was lower in NSCLC tissues and it is a negative regulator of METTL3. MiR-33a can directly target to the 3'UTR of METTL3 mRNA to decrease its expression and further suppress proliferation of NSCLC cells [84]. Therefore, more and more evidences show that the interaction between miRNAs and m6A modification can affect the development of cancer. However, the detailed mechanisms of these regulatory functions were not fully understood.

### m6A modification and circRNAs

#### The role of m6A modification in circRNAs

The m6A modification in linear RNA is comprehensive because m6A can regulate various aspects of the modified RNA including transcription, alternative splicing, advanced structure, nuclear translocation, stability and translation [17, 22, 56, 85]. Similar findings were also observed in the m6A-modified circRNAs. m6A-modified circRNAs frequently derive from unmethylated exons of certain mRNAs. It has already been confirmed that m6A is widespread in circRNAs and exhibits difference compared to mRNA-m6A-modifications. These differences include the following aspects: a. The same position in the linear RNA corresponding to m6A-modified circRNAs is rarely modified by m6A, and mRNA is more likely to carry m6A modification in its 3'UTR region. b. m6A-modified circRNAs exhibit a high cell-specific distribution, and the same circRNA molecule has different m6A-modified states in different cells. c. m6A-modified circRNAs often correspond to large exon regions, and transposition elements tend to be present on both sides. d. CircRNAs and linear RNAs have modification and recognition enzymes in common. e. The mRNA corresponding to m6A-modified circRNAs is relatively more unstable [86].

The m6A sites in circRNAs can act as an internal ribosomal entry site (IRES) to drive translation initiation. Previous studies reported that the presence of IRES elements in circRNAs may facilitate subsequent frame translation [87]. Moreover, it has confirmed that m6A modification can not only initiate the translation of certain circRNAs, but also affect their translation efficiency. Further analyses showed that m6A-driven translation of circRNAs is widespread, with hundreds of endogenous circRNAs having translation potential. This expands the field of coding of the human transcriptome and proposes the role of circRNA-derived proteins in the response of cells to environmental stress [88].

The degradation of circRNA is of importance to understand its function and expression, but the related mechanism is very limited. It has shown that YTHDF2-mediated degradation of m6A-modified RNA is effective not only for linear mRNAs, but also for circRNAs, greatly improving the understanding of the mechanism of circRNA degradation [89]. Unlike linear mRNA with a 5′ cap and a 3′ poly(A) tail, circRNAs are resistant to degradation by exoribonucleases and can only be degraded by endoribonucleolytic cleavage. Recent study has shown that some m6A-containing circRNAs undergo m6A-meditated pathway of YTHDF2–HRSP12–RNase P/MRP-mediated rapid endoribonucleolytic degradation [90].

Recent study has shown that m6A modification can also regulate the generation of ORF-carrying circRNAs during the development of mouse male germ cells, which benefits to understand the function of m6A modification in circRNAs [91]. YTHDC1 was reported to interact with circNSUN2 to promote the cytoplasmic output of circNSUN2. In the exon 5-exon 4 junction site of circNSUN2, YTHDC1 can bind to the m6A-binding motif at the GAACU m6A motif in circNSUN2, which facilitates circNSUN2 exporting from the nucleus to the cytoplasm by an m6A-dependent manner [92].

In summary, with the increasing research on circRNAs and m6A modification, the regulation of circRNAs by m6A modifications has become apparent, and the associated regulatory mechanisms have been confirmed to play crucial roles in the biological function of cells.

#### The role of m6A modification in circRNAs in tumor

As a functional molecule, circRNAs are of great significance in the process of life activities and even some pathogenesis [91]. Recently, studies have shown that circRNAs play crucial roles in the occurrence, development and metastasis of cancer.

m6A-modified circRNAs have been implicated in tumor metastasis by regulating the stability of mRNA. m6A-modified circNSUN2 is shown to be associated with liver metastasis in colorectal cancer and predicts poor patient survival. Researchers have found that circNSUN2 is frequently up-regulated in serum samples from patients with CRC liver metastasis (LM). m6A-modified circNSUN2 increased the cytoplasmic output, and circNSUN2 enhanced the stability of HMGA2 mRNA by forming the RNA-protein ternary complex of circNSUN2 / IGF2BP2 / HMGA2 in the cytoplasm, which promotes CRC metastasis process [92]. These findings clarify that m6A-modified circRNAs can regulate cytoplasmic output and stabilize mRNA to promote cancer metastasis, and suggest that circRNAs may be an important prognostic marker and therapeutic target for the cancer.

Not only are circRNAs regulated by m6A, but m6A can also be regulated by circRNAs to promote the progression of cancer. CircRNAs can regulate m6A modification by the form of sponge adsorbed miRNAs, thus affecting tumor progression. In the kidney renal clear cell carcinoma (KIRC), the mRNA expression level of METTL14 in KIRC tissues was significantly reduced, which was negatively correlated with KIRC stage. By using bioinformatics tools to establish an interaction network, a total of 24 circRNAs was predicted to interact with miR-130a-3p, miR-130b-3p, miR-106b-5p and miR-301a-3p. Those circRNAs can regulate the mRNA of METTL14 as miRNAs sponge and further regulate the expression of PTEN mRNA, a known tumor suppressor gene, by changing its m6A RNA modification level [93].

In summary, circRNAs regulation by m6A modifications has important influence on cancer development. Exploring the effects of m6A modification on key circRNAs will help elucidate the comprehensive role of circRNAs in tumors. This provides a novel direction to explore the complex epigenetic regulation network of organisms, which also provides an important theoretical basis for the regulation and target of tumor.

## Conclusion and perspectives

**Fig. 2:**
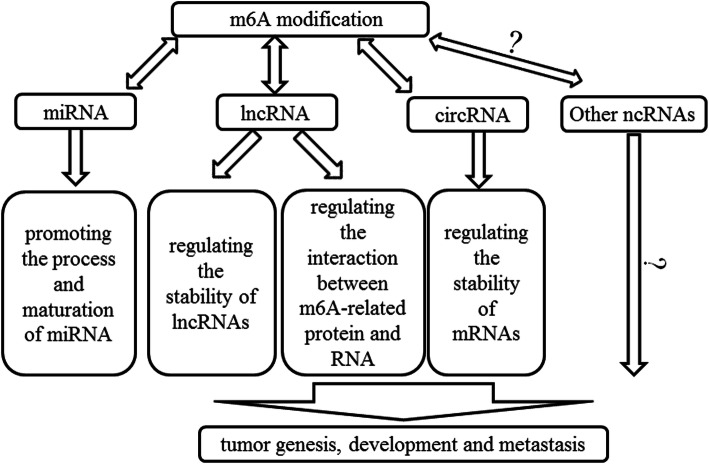
The summary of the interaction between m6A and ncRNAs in cancer. In cancer cells, m6A modification could regualte the stability lncRNAs or recruit the m6A recogenizing protein to lncRNAs. m6A modification could also affect the process and maturation of miRNAs. As for circRNAs, m6A could regulate the stability of mRNA through circRNAs. These ncRNAs are all involved in the regulation of m6A methylation process

Here we reviewed the function of m6A methylation in lncRNA, miRNA and circRNA (Fig. 2). Someother ncRNAs can also be m6A methylated, such as ribosomal RNA (rRNA) and small nuclear RNA (snRNA). There are two m6A sites located at the functionally important region of human rRNAs, including position 1832 of 18S rRNA and position 4220 of 28S rRNA, which may be important for the translation and ribosome heterogeneity [94–96]. The methylation of rRNAs is potentially associated with ribosomopathies, which are often involved in tumorigenesis [97]. In addition, A43 of the highly conserved ACAGAGA box of U6 snRNA is also m6A methylated, which may affect the base pairs with the 5′ splice site of mRNA during pre-mRNA splicing [16, 98, 99]. Small nucleolar RNAs (snoRNAs) are also found that they possess m6A methylation sites [100]. However, the specific impact of the m6A methylation on the physiologic function of snoRNAs still needs further investigation. Therefore, m6A methylation do widely present in various ncRNAs and more efforts should be contributed to reveal the regulation system of the methylation of ncRNAs.

NcRNAs are highly abundant in the human genome, and they regulate multiple biological processes at the level of transcription, translation, and post-translation. Since ncRNAs can promote the occurrence and development of tumors, their research has received extensive attention. NcRNAs have been shown to have abundant m6A modification sites. Benefiting from the advancement of novel technologies and bioinformatics analysis, a growing number of m6A-modified ncRNAs have been identified, annotated and functionally predicted. The high-throughput sequencing developed these years has been used in studies associated with m6A and ncRNAs based on m6A antibody immunoprecipitation with or without crosslinking [101, 102]. However, these methods cannot identify precise m6A sites because of the non-specific recognition of the antibody and the mapping window or varied distance from the m6A sites. Recently, several antibody-free approaches are established to detect the m6A modification at single base resolution. One antibody-independent enzymatic method, also named as m6A-REF-seq or MAZTER-seq, takes advantage of RNA ACA motif-sensitive RNase cleavage, thus representing about 16% of total m6A sites [103, 104]. Another antibody-free method, called DART-seq, makes use of the fusion construct of YTH domain with the cytidine deaminase APOBEC1 to induce C-to-U deamination at sites adjacent to m6A sites, which can be detected using standard RNA sequencing [105]. A metabolic labeling method using Se-allyl-l-selenohomocysteine to substitute the methyl group on the enzyme cofactor SAM with the allyl was also developed to detect m6A sites [106]. Other new technologies are also expected to promote new understanding of m6A modification of ncRNAs, such as third-generation sequencing technology, newly designed mass spectrometry protocols technology and improved chromatography [107]. This will make it more and more convenient to study the relationship between m6A and ncRNAs.

Despite research methods of m6A regulation of ncRNAs are gradually improved, there are still some problems to be solved. Firstly, unlike mRNA, ncRNAs cannot be enriched by poly(A) tail, which means it is really difficult to obtain pure lncRNAs or circRNAs. Secondly, these methods detecting m6A modification can barely differentiate closely deposited m6A clusters from each other, which limits the researchers to quantify the methylation level of certain transcripts in different treatment groups. Last but not least, the m6A modification is dynamic and can be regulated by multiple factors. This makes the result of high throughput sequence of m6A sites unstable and hard to be repeatable. A good example of this is the controversy about the ability to remove m6A in vivo by FTO. More efforts should be devoted to explore the fundamental system of m6A.

On the other hand, the interaction between ncRNAs and m6A can regulate multiple biological processes, and play a key role in the occurrence and development of tumors. Most studies have shown the abnormally expressed m6A-related protein affects the level and function of ncRNAs and further affects tumor development. There are also evidences shown that ncRNAs can regulate m6A modification and influence the expression of cancer genes. ncRNAs facilitate the interaction between m6A methylase (or m6A demethylase, m6A methylation recognition protein) and target mRNA nascent transcripts and induce the methylation (or demethylation, m6A methylation recognition) of pre-mRNA, which increases the expression of target mRNA and influences the progression of cancer [69, 70]. miRNAs can directly target to the 3'UTR of METTL3(or YTHDF2, FTO) mRNA to decrease its expression and further suppress proliferation of tumor cells [78, 84]. Specifically, there are many studies on the function of m6A-modified circRNAs, but there are only few reports of m6A-modified circRNAs in tumor, and further research is needed.

m6A-modified ncRNAs have great potential in clinical applications. At present, although many of ncRNAs have been selected as good biomarkers for early cancer detection or drug response, there is a wide gap away from clinical applications. RNA modification has been selected as a crucial strategy. It has been reported that the regulators or inhibitors of the m6A may have therapeutic potential in cancer. The ethylester form of meclofenamic acid 2 (MA2) has been shown to effectively suppresses GBM progression effectively by inhibiting FTO and R-2HG has antileukemic effects by increasing m6A modification in sensitive cells via suppressing FTO [108, 109]. According to our previous description, m6A-modified ncRNAs are related to the biological behaviors of cancer, such as enhancing the stemness of cancer cells, promoting the growth and proliferation of cancer cells, and increasing anti-radiotherapy or chemotherapy [59]. The abnormal expression of ncRNAs mediated by m6A modification helps certain activation of oncogenes or tumor suppressor genes, and plays a key role in the occurrence and development of tumors [81]. Therefore, we can target m6A modification to regulate ncRNAs, and thus influencing the development of cancer.

In summary, the correlation between m6A modification and ncRNAs provides a new perspective for exploring the potential regulatory mechanism of tumor gene expression. We believe it is necessary to further understand the molecular mechanism of the interaction between ncRNAs and m6A modification, and the mechanism of mutual regulation in other tumors. This makes it possible for ncRNAs and m6A to be used as cancer indicators and even as targets for future treatment.

## Data Availability

Not applicable.

## Abbreviations

ncRNAs: Noncoding RNAs
lncRNAs: Long noncoding RNAs
miRNAs: MicroRNAs
circRNAs: Circular RNAs
m6A: N6-methyladenosine
METTL: Methyltransferase-like protein
ALKBH5: AlkB homolog 5
FTO: Fat-mass and obesity associated protein
YTHDF: YTH domain family
YTHDC: Human YTH domain containing
HNRNP: Heterogeneous nuclear ribonucleoprotein proteins
AML: Acute myeloid leukemia
HAT: Histone acetyltransferase
MREs: miRNA response elements
ceRNA: Competing endogenous RNAs
RBP: RNA binding protein
MALAT1: Metastasis-associated lung adenocarcinoma transcript 1
PVT1: Plasmacytoma Variant Translocation 1
HNSCC: Head and neck squamous cell carcinoma
HCC: Hepatocellular carcinoma
HDGF: Hepatoma-derived growth factor
NSCLC: Non-small-cell lung cancer
GSC: Glioblastoma stem-like cells
CC: Cervical cancer
pre-miRNA: Precursor miRNA
IRES: Internal ribosomal entry site
KIRC: Kidney renal clear cell carcinoma
